# Salvage Pneumonectomy for Rapidly Progressive Destroyed Lung during Durvalumab Maintenance after Chemoradiotherapy

**DOI:** 10.5761/atcs.cr.26-00053

**Published:** 2026-06-10

**Authors:** Shin-nosuke Watanabe, Daisuke Kimura, Yoshiaki Saito, Kageaki Taima, Masahiko Aoki, Akira Kurose, Masahito Minakawa

**Affiliations:** 1 Department of Thoracic and Cardiovascular Surgery, Hirosaki University Graduate School of Medicine, Hirosaki, Aomori, Japan; 2 Department of Respiratory Medicine, Hirosaki University Graduate School of Medicine, Hirosaki, Aomori, Japan; 3 Department of Radiation Oncology, Hirosaki University Graduate School of Medicine, Hirosaki, Aomori, Japan; 4 Department of Anatomic Pathology, Hirosaki University Graduate School of Medicine, Hirosaki, Aomori, Japan

**Keywords:** durvalumab, destroyed lung, salvage pneumonectomy, aspergillosis, frozen hilum

## Abstract

Durvalumab maintenance therapy following chemoradiotherapy is the standard treatment for patients with unresectable stage III non–small-cell lung cancer. Immune checkpoint inhibitors can alter host immunity, potentially amplifying inflammatory responses during opportunistic infections. We report a rare case of rapidly progressive destroyed lung caused by invasive pulmonary aspergillosis during durvalumab therapy, which was successfully managed with salvage pneumonectomy. A 53-year-old man with locoregionally recurrent lung adenocarcinoma received chemoradiotherapy followed by durvalumab treatment. Invasive pulmonary aspergillosis developed on day 74. Despite intensive antifungal therapy and corticosteroid administration, the right lung developed extensive liquefactive necrosis beyond the irradiated field within 5 weeks. Given the risk of fatal hemoptysis and sepsis, an emergency right pneumonectomy was performed. The frozen hilum required intrapericardial vascular control and reinforcement of the bronchial stump. Recovery was uneventful, and pathological examination confirmed aspergillosis without residual malignancy. Prompt recognition of medical refractoriness and timely surgery are critical for survival.

## Introduction

Durvalumab maintenance therapy following chemoradiotherapy (CRT) is currently the established standard of care for unresectable stage III non–small-cell lung cancer (NSCLC).^1)^ With its widespread adoption, the management of immune-related adverse events (irAEs) has become a critical clinical challenge.^2)^ Recent reports suggest that immune checkpoint inhibitors (ICIs) may modulate the host inflammatory response to infections, potentially triggering destructive lung injuries that are difficult to explain within the conventional framework of radiation pneumonitis or ICI-induced pneumonitis. Specifically, cases wherein opportunistic infections during durvalumab therapy cause rapid and irreversible parenchymal destruction and a destroyed lung are exceedingly rare, and no definitive treatment strategies have been established. Salvage pneumonectomy in this context is technically demanding because prior CRT and ongoing inflammation often result in dense fibrosis and a frozen hilum. Here, we report a case of rapidly progressive destroyed lung triggered by an invasive fungal infection during durvalumab maintenance, which was successfully treated with a life-saving right pneumonectomy. This case provides valuable insights into the pathophysiology of severe lung injury following CRT and ICI therapy and highlights the role of surgical intervention in extreme clinical scenarios.

## Case Report

A 53-year-old man with a history of chronic obstructive pulmonary disease (COPD), dyslipidemia, and angina pectoris, as well as a smoking history of 28 pack-years, had previously undergone a right lower lobectomy with ND2a lymph node dissection for lung adenocarcinoma. The pathological stage was IIIA (pT1bN2M0, according to the eighth edition of the Union for International Cancer Control Tumor-Node-Metastasis classification), and companion diagnostics revealed a KRAS G12C mutation and a PD-L1 tumor proportion score of 35%. Following 4 courses of adjuvant chemotherapy with carboplatin and gemcitabine, the patient remained under routine follow-up care. However, 3.5 years after surgery, follow-up computed tomography (CT) revealed recurrence in the hilar and mediastinal lymph nodes at stations 10, 4R, and 5 (**Figs. 1A** and **1B**). Pre-treatment positron emission tomography CT (PET-CT) demonstrated increased fluorodeoxyglucose uptake corresponding to these enlarged lymph nodes. A multidisciplinary conference involving pulmonology, radiation oncology, and thoracic surgery determined that CRT was appropriate according to the protocol for stage III NSCLC. Chemotherapy consisted of 2 cycles of cisplatin (60 mg/m^2^) and S-1 (80 mg/m^2^/day). Radiation therapy was administered to the recurrent hilar and mediastinal lymph nodes using a conventional schedule of 60 Gy in 30 fractions (**Figs. 1C** and **1D**). Dose-volume constraints were within generally accepted limits: the lung V20 was 29%, and the maximum dose to the spinal cord was 44 Gy. Following CRT, durvalumab was maintained at 1500 mg every 4 weeks. Although PET-CT was not repeated following CRT and durvalumab therapy, follow-up CT showed complete resolution of all previously involved lymph nodes. On day 74, following 3 cycles, the patient developed fever and cough, and new infiltrative shadows appeared in the right lung on chest radiography and CT (**Fig. 2A**). Durvalumab was discontinued, and the patient was admitted with suspected grade 2 immune-related pneumonitis or bacterial pneumonia. Treatment with prednisolone (60 mg/day) and meropenem was initiated. Laboratory tests showed a mildly elevated serum KL-6 level, while Aspergillus galactomannan antigen and beta-D-glucan were positive. Bronchoscopy was deferred because of the risk of hemorrhage and respiratory failure, particularly given the positive *Aspergillus* antigen and the detection of *Stenotrophomonas maltophilia* in sputum cultures. Antimicrobial therapy was escalated to include antifungal treatment; however, the right lung shadows rapidly worsened. CT on day 5 after admission showed liquefactive necrosis and cavitation (**Fig. 2B**), which progressed to a destroyed lung by day 22 (**Fig. 2C**). By day 36, new infiltrates appeared in the left lung, suggesting contralateral spread of the infection (**Fig. 2D**). Despite preserved oxygenation (SpO_2_ >90% on room air), the risks of further progression, hemorrhage, and sepsis were considered critically high. The multidisciplinary team concluded that medical management had reached its limits, and an emergency salvage right pneumonectomy was planned.

**Fig. 1:**
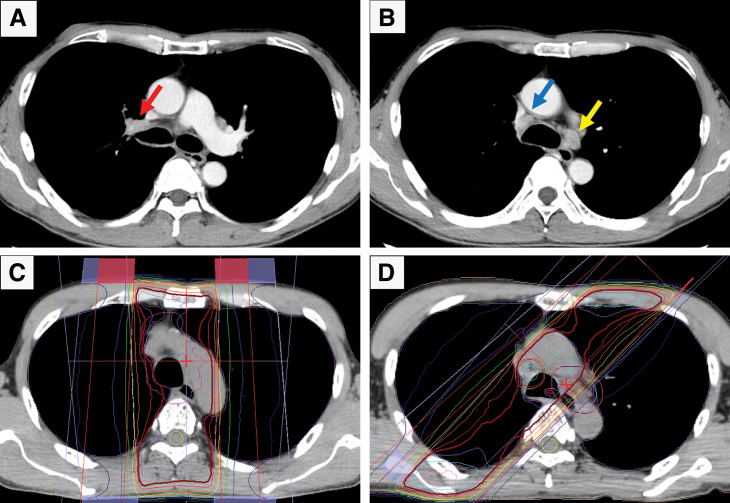
Mediastinal and hilar lymph node recurrence and radiation therapy planning. (**A**, **B**) Contrast-enhanced chest computed tomography at the time of recurrence, showing lymphadenopathy at stations 10 (red arrow), 4R (blue arrow), and 5 (yellow arrow). (**C**, **D**) Radiation treatment planning and dose distribution maps. The high-dose field was localized to the hilar and mediastinal regions, with lung V20 maintained within acceptable limits (29%).

**Fig. 2:**
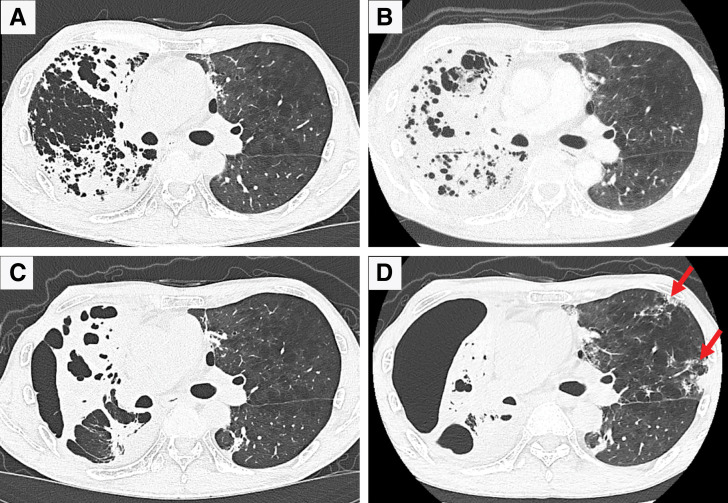
Serial chest computed tomography scans showing rapid progression of the destroyed lung. (**A**) At the time of admission, new infiltrative shadows were evident in the right residual lung following durvalumab maintenance therapy. (**B**) By post-admission day 5, progressive liquefactive necrosis and multiple cavitary lesions had developed. (**C**) By post-admission day 22, extensive parenchymal destruction and large cavity formation were observed, representing the radiological features of a destroyed lung. (**D**) On post-admission day 36, the right lung remained destroyed. Red arrows indicate new infiltrative shadows in the left lung, suggesting the spread of infection or inflammatory spillover.

Surgery was performed under general anesthesia with 1-lung ventilation while the patient was in the supine position. Vascular access was secured for potential venoarterial extracorporeal membrane oxygenation (ECMO) support in case of intraoperative instability. Access was achieved via a right anterolateral thoracotomy through the fourth intercostal space (**Fig. 3A**). To ensure adequate exposure, the internal thoracic vessels were ligated, and the fourth rib was transected near the sternum. The residual right lung, consisting of the upper and middle lobes, exhibited dense circumferential fibrous adhesions to the chest wall and mediastinum. During dissection, purulent fluid was collected and sent for culture. The lung parenchyma was extremely friable due to liquefactive necrosis, posing a high risk of rupture and contamination. The hilum was markedly fibrotic owing to the combined effects of radiation and inflammation, resulting in a frozen hilum. Because safe dissection and vascular identification from the pleural side were impossible, we prioritized safety and opted for an intrapericardial approach. The superior pulmonary vein and main pulmonary artery were carefully dissected and encircled within the pericardium and then safely transected using a linear stapler (**Fig. 3B**). The main bronchus was densely adherent to the surrounding tissue, showing woody induration due to post-radiation scarring, which made it difficult to distinguish the cartilaginous rings. Following sharp dissection of the dense intrathoracic pleural adhesions involving the lung, the right main bronchus was identified and transected using a thick-tissue linear stapler (**Fig. 3C**). Mediastinal lymph node dissection was considered; however, the mediastinum was severely fibrotic due to prior CRT and ongoing inflammation. Given the absence of radiological evidence of residual nodal disease and concerns regarding operative safety, lymph node dissection was not performed. After thorough irrigation, an intercostal muscle flap was harvested and used to reinforce the bronchial stump (**Fig. 3D**). The pericardial defect was reconstructed using a prosthetic sheet. The operative time was 6 h and 7 min, with an estimated blood loss of 1210 mL. ECMO was not required because the patient remained hemodynamically stable throughout the intraoperative period. The resected right lung weighed 615 g. The postoperative course was uneventful, with no Clavien–Dindo grade II or higher complications.^3)^ CT on postoperative day 23 confirmed the resolution of the infiltrative shadows in the left lung.

**Fig. 3:**
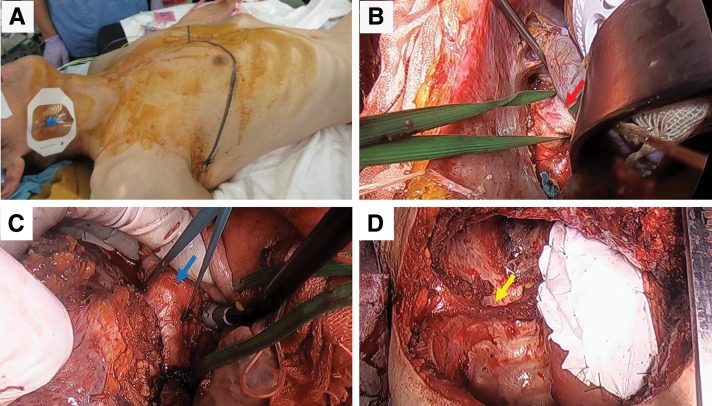
Intraoperative findings of the salvage right pneumonectomy. (**A**) Surgical approach via a right anterolateral thoracotomy. The fourth rib was transected near the sternum to provide adequate exposure of the thoracic cavity and mediastinum. (**B**) Intrapericardial control of the hilar vessels. The right main pulmonary artery (red arrow) is taped laterally to the superior vena cava for safe transection, bypassing the frozen hilum. (**C**) Identification and taping of the right main bronchus (blue arrow) before transection with a thick-tissue linear stapler. (**D**) View of the thoracic cavity following specimen removal. The bronchial stump is reinforced and covered with a pedicled intercostal muscle flap (yellow arrow). Pericardial reconstruction with a prosthetic sheet is also visible.

Pathological examination of the resected right lung revealed severe apical fibrosis and extensive cavitation containing necrotic material, which are characteristic of a destroyed lung. The visceral pleura exhibited diffuse fibrotic thickening (**Figs. 4A** and **4B**). Histologically, the upper lobe exhibited dense inflammatory cell infiltration and fibrosis with fibroblast proliferation (**Fig. 5A**). The cavities contained necrotic tissue with radial and sheaf-like crystals suggestive of calcium oxalate crystals, which are characteristic of *Aspergillus* infection (**Fig. 5B**). The middle lobe showed organizing changes, such as Masson bodies and cholesterol clefts, which were interpreted as secondary changes from the surrounding inflammation (**Fig. 5C**). Grocott staining revealed numerous septate hyphae with acute-angle branching within the necrotic tissue, consistent with *Aspergillus* species (**Fig. 5D**). Although the intraoperative cultures were negative, the histological findings confirmed the diagnosis of pulmonary aspergillosis. There was no evidence of tumor recurrence.

**Fig. 4:**
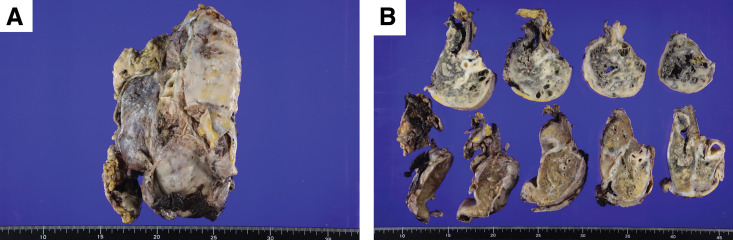
Macroscopic findings of the resected specimen. (**A**) External view of the resected right residual lung (upper and middle lobes). The visceral pleura shows marked, diffuse fibrotic thickening. (**B**) Serial sections of the specimen reveal extensive parenchymal destruction, confirming the macroscopic diagnosis of a destroyed lung.

**Fig. 5:**
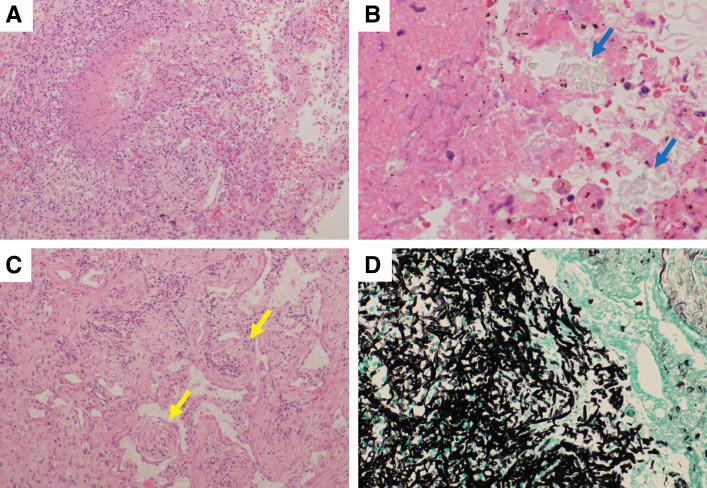
Microscopic findings of the resected right lung. (**A**) Hematoxylin and eosin (H&E) staining shows dense inflammatory cell infiltration and marked fibrosis accompanied by fibroblast proliferation (×100). (**B**) Degenerated and necrotic tissues within the cavity, featuring radial and sheaf-like crystal precipitates (blue arrows), suggestive of calcium oxalate crystals (H&E, ×400). (**C**) Organizing changes such as Masson bodies (yellow arrows) are observed in the middle lobe, representing secondary changes due to the spread of surrounding infection and inflammation (H&E, ×100). (**D**) Grocott staining reveals numerous septate hyphae with acute-angle branching within the cavity and necrotic tissues, consistent with *Aspergillus* species (×400). H&E, hematoxylin and eosin

## Discussion

This case represents an extremely rare instance of opportunistic infection during durvalumab maintenance therapy following CRT-triggered rapid and irreversible parenchymal destruction, leading to a destroyed lung. Although durvalumab has become the standard of care for stage III NSCLC following the PACIFIC study,^1)^ the management of radiation pneumonitis and irAEs remains a significant challenge.^2)^ The distinctive feature of this case is the intersection of radiation-induced tissue damage, an ICI-modulated immune environment, and invasive fungal infection. Initially, the clinical team suspected radiation pneumonitis; however, the shadows appeared in regions entirely distinct from the radiation field, and the rapid liquefactive necrosis was inconsistent with typical radiation injury. Furthermore, when ICI-induced pneumonitis was considered, the localized nature of the initial lesions and the lack of response of the necrotic process to high-dose steroids further distinguished this from conventional irAEs. Given the resistance to general antibiotics, pulmonary aspergillosis was diagnosed, likely facilitated by the patient’s underlying COPD and prior lung injury caused by radiotherapy and ICI. A definitive microbiological diagnosis was not obtained before surgery because bronchoscopy was considered high risk owing to extensive necrosis and the potential for massive hemorrhage and respiratory deterioration. We hypothesized that the immune environment was significantly modulated by durvalumab, leading to a pathology resembling immune reconstitution inflammatory syndrome.^4,5)^ Taima et al. previously suggested that ICIs could excessively amplify inflammatory responses to infections.^6)^ The patient’s clinical response to steroids supported this hypothesis; although the infection was treated with appropriate antifungal agents, the respiratory status remained highly steroid dependent. The rapid deterioration of the left lung upon tapering of steroids, followed by stabilization after re-escalation, strongly suggested that the primary driver of the destruction was an uncontrolled, ICI-mediated hyperinflammatory response to the infection. The patient’s genomic and immunological profiles may have predisposed him to this robust reaction. KRAS G12C-mutated NSCLC is frequently associated with an inflamed tumor microenvironment and elevated PD-L1 expression, suggesting enhanced immunogenicity.^7,8)^ Additionally, the mild elevation in serum KL-6, despite extensive parenchymal destruction, suggests that the primary process was not diffuse type II alveolar epithelial injury but rather infection-triggered, localized necrotic inflammation. Given the failure of medical management to halt this inflammatory cascade, surgical intervention to resect the infectious focus was essential to prevent fatal complications such as contralateral spillover, sepsis, or massive hemoptysis.

Salvage pneumonectomy for refractory infections following CRT and ICI therapy is an exceptionally high-risk procedure because radiation injury, chronic inflammation, and active infection coexist.^9)^ In this context, dense fibrosis often obliterates the normal anatomical planes at the pulmonary hilum, a condition known as a frozen hilum, which necessitates a strategic surgical approach.^10,11)^ Furthermore, the friable nature of the necrotic lung parenchyma requires delicate handling to prevent intraoperative contamination. In this case, the choice of the surgical approach and an early decision on the vascular control strategy were the most critical factors for ensuring a safe outcome. Although median sternotomy is sometimes preferred for inflammatory lung disease to gain early vascular access, we chose a right fourth intercostal anterolateral thoracotomy. This approach provided the necessary exposure for extensive pleurectomy and decortication while allowing harvest of a pedicled intercostal muscle flap from the same operative field. Anticipating potential circulatory collapse, we also simulated a rapid transition to a clamshell incision. Technically, the most pivotal decision was moving to intrapericardial vascular control early during the procedure. Abandoning the attempt to dissect the frozen hilum from the pleural side avoided the risk of massive hemorrhage.^12)^ Additionally, the bronchus exhibited significant woody hardening. The use of a stapler designed for thick tissue, together with reinforcement using a vascularized intercostal muscle flap, was considered important to reduce the risk of bronchopleural fistula, particularly in high-risk situations such as pneumonectomy following CRT. ^13)^

The success of such high-risk surgeries depends not only on surgical skill but also on comprehensive multidisciplinary risk management. The immediate availability of ECMO via preplaced vascular sheaths provides a critical safety net against potential respiratory or circulatory collapse, allowing the surgical team to focus on complex dissection. Safe completion of salvage pneumonectomy for refractory infectious destroyed lung requires an integrated strategy: selecting an appropriate approach for adhesion management, transitioning early to intrapericardial control of the frozen hilum, selecting proper devices, protecting the bronchial stump in hardened tissues, and ensuring a robust perioperative safety system.

## Conclusion

Infection during durvalumab maintenance therapy following CRT can trigger an amplified inflammatory response, potentially leading to rapid progression to a destroyed lung. When medical management fails to control such destructive lung injury, surgical intervention may be a life-saving option, provided that the patient’s general condition is adequate. In salvage pneumonectomy involving a frozen hilum, early intrapericardial vascular control, definitive bronchial stump protection, and multidisciplinary perioperative management are pivotal strategies for achieving a successful outcome.

## Declarations

### Data availability

Data sharing is not applicable to this article, as no datasets were generated or analyzed in the current study.
